# HTLV-1 clonality during chronic infection and BLV clonality during primary infection

**DOI:** 10.1186/1742-4690-8-S1-A185

**Published:** 2011-06-06

**Authors:** Nicolas A Gillet, Carol Hlela, Tine Verdonck, Eduardo Gotuzzo, Daniel Clark, Sabrina Rodriguez, Nirav Malani, Anat Melamed, Niall Gormley, Richard Carter, David Bentley, Charles Berry, Frederic D Bushman, Graham P Taylor, Luc Willems, Charles R M Bangham

**Affiliations:** 1 Department of Immunology, Wright-Fleming Institute, Imperial College London, London, W2 1PG, UK; 2 Molecular and Cellular Epigenetics, Interdisciplinary Cluster for Applied Genoproteomics (GIGA) of University of Liège (ULg), Liège, 4000, Belgium; 3 Instituto de Medicina Tropical Alexander von Humboldt, Universidad Peruana Cayetano Heredia, Lima, Peru; 4 Department of Microbiology, University of Pennsylvania School of Medicine, Pennsylvania, Philadelphia, PA, 19104, USA; 5 Illumina, Chesterford Research Park, Essex, Little Chesterford, CB10 1XL, UK; 6 University of California, California, La Jolla San Diego, CA, 92093-0901, USA; 7 Department of Genitourinary Medicine and Communicable Diseases, Wright-Fleming Institute, Imperial College London, London, W2 1PG, UK

## 

HTLV-1 persists by driving clonal proliferation of infected T-lymphocytes. A high proviral load predisposes to the inflammatory and malignant diseases associated with HTLV-1. Yet the reasons for the remarkable variation within and between individuals in the abundance of HTLV-1-infected clones remain unknown. We demonstrate that negative selection dominates during chronic infection, favouring establishment of proviruses integrated in transcriptionally silenced DNA: this selection is significantly stronger in asymptomatic carriers. We postulated that this selection occurred mainly during the primary infection. We are testing this hypothesis in an animal model by studying the BLV clonality during the primary infection in cows. By measuring the proviral load, the anti-BLV immune response and the BLV clonality we aim to quantify the impact of the immune response on the rate of infectious spread and on the selection of proviruses inserted in a particular genomic environment. Co-infection with Strongyloides stercoralis or Staphylococcus appears to be another risk factor for the development of HTLV-1 associated diseases. We observed that HTLV-1 clonality is altered by co-infection with these pathogens with an increase of both the number and the abundance of the infected T-cell clones. The genomic characteristics of the proviral integration sites in the most abundant clones differ significantly between co-infected individuals and those with HTLV-1 alone, implying the existence of different selection forces in co-infected patients. The rate of appearance of new clones in patients co-infected with Strongyloides stercoralis is higher than in patients with HTLV-1 alone. By comparing skin lesions and blood samples from patients with Infective Dermatitis associated with HTLV-1 (IDH), we observed a significant proportion of distinct infected clones between the two compartments. The skin lesions seem to be a site for HTLV-1 infectious spread.

